# Ex vivo modelling of the formation of inflammatory platelet-leucocyte aggregates and their adhesion on endothelial cells, an early event in sepsis

**DOI:** 10.1007/s10238-018-0526-1

**Published:** 2018-09-06

**Authors:** Azzah Alharbi, Jonathan P. Thompson, Nicholas P. Brindle, Cordula M. Stover

**Affiliations:** 1 Department of Infection, Immunity and Inflammation, College of Life Sciences, University of Leicester, Leicester, LE1 9HN UK; 2 King Abdulaziz University, Jeddah, Saudi Arabia; 3 Department of Cardiovascular Sciences, Division of Anaesthesia, Critical Care and Pain Management, University of Leicester, Robert Kilpatrick Clinical Sciences Building, Leicester Royal Infirmary, Leicester, LE2 7LX UK; 4 Department of Cardiovascular Sciences, College of Life Sciences, University of Leicester, Leicester, LE1 9HN UK; 5 Department of Molecular & Cell Biology, College of Life Sciences, University of Leicester, Leicester, LE1 9HN UK

**Keywords:** Whole blood, Hirudin, Platelet-leucocyte aggregates, Sepsis, Endothelial adhesion, Bacterial

## Abstract

Septicaemia is an acute inflammatory reaction in the bloodstream to the presence of pathogen-associated molecular patterns. Whole blood stimulation assays capture endotoxin-induced formation of aggregates between platelets and leucocytes using flow cytometry. We wanted to assess extent of spontaneous aggregate formation in whole blood stimulation assays and compare the effects of endotoxin and heat-killed, clinically relevant, bacterial pathogens on aggregate formation and then on adhesion of aggregates to TNFα-stimulated endothelial cells. We found that endotoxin (from *Escherichia coli* or *Salmonella enteritidis*) was not a suitable stimulus to provoke platelet-leucocyte aggregates in vitro, as it did not further increase the extent of aggregates formed spontaneously in stasis of hirudin-anticoagulated blood. Specifically, whole blood samples stimulated with or without LPS produced aggregates with a mean surface area of 140.97 and 117.68 μm^2^, respectively. By contrast, incubation of whole blood with heat-killed *Klebsiella pneumoniae* or *Staphylococcus aureus* produced significantly enhanced and complex cellular aggregates (with a mean surface area of 470.61 and 518.39 μm^2^, respectively) which adhered more frequently to TNFα (and free fatty acid)-stimulated endothelial cells. These were reliably captured by scanning electron microscopy. Adhesion of cellular aggregates could be blocked by incubation of endothelial cells with a commercial P-selectin antibody and an angiopoietin-2 ligand trap. In conclusion, we have developed an in vitro method that models the acute inflammatory reaction in whole blood in the presence of sepsis-relevant bacterial pathogen surfaces.

## Introduction

Sepsis is caused by a dysregulated host response to infection with bacterial, viral or fungal pathogens and may lead to life-threatening organ dysfunction, affecting in particular: gut, lung, kidney, heart and brain. It constitutes a global health problem, accounting for 31.5 million cases worldwide each year, with an associated 5.3 million deaths [1]. Despite improvements in medical care, sepsis remains a serious condition with high mortality. The lack of biomarkers that trace the extents of the immune response in the course of sepsis (immune activation, over activation and exhaustion during development and progression of sepsis) poses a problem in the design of new treatment approaches [2, 3]. Platelet-leucocyte aggregates, whether they are circulating in blood or adhering to the endothelium, are extensively formed in sepsis, play a critical role in sepsis pathophysiology and correlate with severity [4, 5]. Molecular interactions that lead to the formation of these cellular aggregates and their adherence to endothelial cells could represent novel targets to use for therapy.

A recently conducted retrospective study of admissions to intensive care units showed bacterial sepsis in 60% of all 172 cases. Approximately two-thirds were diagnosed with endotoxemia [6]. The initial antibacterial immune response following infection is triggered by recognition of conserved molecular products of pathogen termed pathogen-associated molecular patterns (PAMPs). Pathogen recognition receptors (PRRs) are expressed by innate immune cells (platelets, monocytes, macrophages and to some extent endothelial cells) and lead to activation of intracellular signalling cascades and production of inflammatory mediators such as TNFα, IL-1, IL-6, IL-12 and IL-8 [7]. Prolonged activation of such signalling pathway leads to an exaggerated inflammatory response, and exhaustion.

Platelets may interact directly with pathogens or their PAMPs [8] and are instrumental in several steps of leucocyte recruitment, activation and adhesion to endothelial cells [9]. Neutrophils attach to activated platelets mainly through P-selectin, a protein expressed on the platelet surface upon activation, via PSGL-1 (P-selectin Glycoprotein Ligand-1). P-selectin and PSGL-1 interaction is critical for tethering and rolling of the neutrophil on the platelet surface, as studies aimed at blocking either of these molecules with a monoclonal antibody resulted in complete inhibition of the initial interaction between platelets and neutrophils [10, 11]. This interaction results in further neutrophil activation and upregulation of other adhesion molecules (integrins) such as macrophage-1 antigen, Mac-1(CD11b/CD18 or complement receptor 3), and lymphocyte function-associated antigen, LFA-1 (CD11a/CD18), that lead to firm adhesion. Mac-1 binds to GPIbα or junctional adhesion molecules-3 (JAM-3) present on the platelet surface [12]. Furthermore, activated platelets express CD40 ligand (CD40L) and shed this into circulation. Platelet-derived CD40L can bind to CD40 expressed on their surface leading to more platelet activation [13] and to neutrophil CD40 [14]. It can also interact with endothelial CD40 leading to stimulation of endothelial cell to upregulate expression of various adhesion molecules, such as ICAM and VCAM, and to release the chemokine, CCL2, thereby promoting recruitment of neutrophils. Additionally, in sepsis, activated platelets can interact with neutrophils through triggering receptor expressed on myeloid cells (TREM1) that leads to further stimulation of neutrophils and increased expression of adhesion molecules [15].

LPS-dependent TLR4 signalling is suggested as a key pathway in the pathogenesis of gram-negative sepsis [16, 17]. In in vivo studies using TLR4 deficient mice, administration of purified LPS failed to induce an immune response. Individuals with *TLR4* polymorphisms were more susceptible to (gram negative) meningococcal sepsis [18]. LPS, known as endotoxin and situated on the outer membrane of gram-negative bacteria, has been widely used to model sepsis event in vivo and in vitro.

The relevant literature describing LPS-induced platelet-leucocyte aggregates in human peripheral venous blood shows a large variability in reported levels of circulating platelet-leucocyte aggregates. There is no consensus regarding anticoagulants, buffers, sample handling and dilution, cell treatment, immune labelling protocols, and cytometer settings. Some studies have isolated platelets and granulocytes from whole blood, mixed them at a certain ratio and incubated the mixture in the presence or absence of LPS, disregarding the possibly important effects of other plasma components, red blood cells and cell activation during sample handling on platelet–leucocyte interactions [19]. Most importantly, however, studies compared the extent of LPS-induced aggregates to conditions where the blood sample was immediately stained, unincubated and unstimulated [20, 21].

Endothelial cells play an essential role in sepsis, as a main target and regulator, in terms of site, extent and duration to ensure an adequate host inflammatory response and resolution [22]. Normally, endothelial cells provide an anti-inflammatory and non-adhesive surface to the blood flow. Their inflammatory activation results in exocytosis of Weibel–Palade bodies and release of components such as P-selectin, von Willebrand factor (vWF) and angiopoietin 2. Increased expression of adhesion molecules such as P- and E-selectin, VCAM and ICAM and of cytokines and chemokines further mediate endothelial cell–leucocyte–platelet interactions [23]. Adhesion of platelet-leucocyte aggregates to the endothelium has been analysed in in vivo models of sepsis [5] and of acute inflammation [24]. However, there is currently no in vitro study that investigates platelet-leucocyte aggregate adhesion to endothelium in a condition that mimics sepsis.

Thus, there is a need for a reliable in vitro model to investigate sepsis-relevant formation of platelet-leucocyte aggregates and their adhesion to activated endothelial cells.

Two different types of LPS were used in this study because they differed in their stimulatory effect for complement activation and we wanted to include analysis of the effect of complement on the aggregate formation. A study aimed at investigating the effect of *E. coli O111:B4* LPS on complement activation found that incubation of lepirudin-anticoagulated blood with *E. coli* LPS did not activate the complement [25]. Ongoing mouse work in our laboratory, aimed at studying the activity of complement in response to LPS, showed that incubating mouse serum with *Salmonella* LPS for 1 h induced complement activation, as was described previously [26].

Clinically important pathogens such as *Klebsiella pneumoniae* and *Staphylococcus aureus* were investigated here as alternative stimuli to LPS because they provide a wide range of sepsis-relevant PAMPs. *Klebsiella pneumoniae*, a gram-negative encapsulated bacterium, is the second most common isolated pathogen in patients with gram-negative sepsis [27, 28]. *S. aureus*, a gram-positive bacterium, is one of the most frequently isolated microorganisms in gram-positive sepsis [29, 30].

Thus, in this study, we investigated the usefulness of LPS vs heat-killed bacteria in generating platelet-leucocyte aggregates and their adherence to activated endothelium. Our aim was to develop a model of the acute inflammatory reaction to common bacterial PAMPs, for future investigation of possible therapeutic agents.

## Materials and methods

### Ethics

Approval for this study was obtained from the University of Leicester Committee for Research Ethics and held by AA (No. 5357). Whole blood samples were taken from a total of 10 healthy adult (female and male) volunteers on two to ten separate occasions after informed and free, written consent. Individuals were between 25 and 45 years, non-smokers, with normal body mass index and free of medication (in particular: non-steroidal anti-inflammatory drugs and aspirin) for at least 3 days. Samples were anonymised.

### Blood sample collection

Whole blood was collected in the morning with light tourniquet by peripheral venous puncture using G21 needle into tubes containing 10 mM ethylenediamine tetra acetic acid (EDTA, pH 8), or Hirudin (150 U/ml) (Merck Millipore, UK), inverted gently to ensure proper mixing of whole blood with anticoagulant and processed strictly within 10 min of collection.

### Bacteria and growth condition

*Klebsiella pneumoniae* (clinical isolate, KR3153) was obtained from a departmental collection at the University of Leicester. Community associated methicillin-resistant *Staphylococcus aureus* (MRSA) was provided by Dr J Morrissey, University of Leicester. All bacteria were grown at 37 °C in Lauria Bertani medium (LB), monitored by reading the optical density at 600 nm till late logarithmic phase, to ensure the maturity of the bacteria and maximal production of surface proteins prior to their shedding (personal communication Dr J Morrissey), suspended in phosphate-buffered saline (PBS). The numbers of colony-forming units (CFU) were quantified by streaking serial dilutions of the suspension onto LB agar plate before heat inactivation (60 °C, 30 min for *K. pneumoniae*; 80 °C, 10 min for *S. aureus).* Success of heat treatment was confirmed by overnight incubation of a streaked suspension on LB agar. An amount equivalent to a final concentration of 10^6^ CFU/ml was used from washed, frozen stocks of heat-inactivated bacteria as stimulus to human whole blood.

### Whole blood stimulation with LPS or heat-killed bacteria

In separate experiments, whole blood was immediately incubated with and without (control) LPS from *E. coli 0111: B4* (Invivogen, Toulouse, France) and from *Salmonella enteritidis* (Hycult Biotech, UK) at 1 μg/ml or heat-killed preparations from *K. pneumoniae* or *S. aureus* (termed HKK and HKS, respectively) at a dose of 10^6^CFU/ml for 1 h at 37 °C. This dose was based on studies in which in vitro whole blood stimulation of heat-killed bacteria was used to investigate the inflammatory response of whole blood in terms of TNF and IL-6 production [31]. All tubes, tips and solutions were endotoxin free to avoid artefactual introduction of endotoxin.

### Flow cytometric analysis of platelet granulocyte aggregates

Aliquots of whole blood were stained, after titration, with 2.5 μg/ml mouse antihuman CD42b: PE (clone HIP1 to detect platelets) and 15 μg/ml mouse antihuman CD66b: APC (clone G10F5 to detect granulocytes) simultaneously or PE mouse IgG 1 and APC mouse IgM as isotype controls. Single stains were also prepared as fluorescence minus one (FMO) control. All antibodies were purchased from Biolegend, UK. After erythrolysis, samples were fixed, stored at 4 °C and analysed by flow cytometry within 24 h of fixation using FACS Aria II Flow Cytometer (BD Biosciences, UK) equipped with FACSDiva software version 6.1.3 (BD Biosciences). After compensation using BD™ CompBeads set Anti-mouse Igκ (BD Biosciences), samples were acquired with a medium flow rate to decrease the coincidence that gives false positive results of platelet granulocyte aggregates using identical flow cytometer experimental setup each time. A minimum of 5000 granulocyte events were acquired for analysis. Events that stained positively for both platelet and neutrophil markers were considered platelet granulocyte aggregates.

### Preparation of samples for electron microscopy of platelet-leucocyte aggregates

For whole blood stimulation assay, the samples were washed (800 *g*, 5 min) after erythrolysis and prepared on 24-well plates prepared with glass cover slips (diameter 13 mm). For the adhesion assay, endothelial cells grown on cover slips were washed after whole blood incubation. Then, all samples were processed as follows: fixation with 2.5% glutaraldehyde in PBS, wash in PBS buffer (3 times 10 min), post-fixation in 1% osmium tetroxide/0.1 M in PBS for 45 min, wash in double-distilled water (3 times 10 min). The samples were next dehydrated in serial ethanol concentrations. For scanning electron microscopy (SEM), the samples were critical point-dried with CO_2_, mounted onto aluminium stubs using carbon sticky tabs and sputter-coated with a 300-Å layer of gold palladium for 90 s 2.2 kV. Then, samples were examined with the Hitachi S3000H scanning electron microscope with an accelerating voltage of 10 kV. For transmission electron microscopy (TEM), the samples were taken through serial concentrations of (25, 50, 75 and 100%) Agar low-viscosity resin in propylene oxide, embedded, and polymerised at 60 °C for 16 h. Samples were sectioned using a Reichert Ultracut S Ultramicrotome and double-stained with 2% uranyl acetate and Reynold’s lead citrate and then viewed on the JEOL 1400 TEM with an accelerating voltage of 100 kV. Images were captured using Mageview III digital camera with iTEM software.

### Cell culture

Immortalised human umbilical vein endothelial cells (EA.hy 926) were kindly provided by Dr. N. Abbassian, Department of Infection, Immunity and Inflammation, University of Leicester. These cells were cultured till confluence in DMEM (Fisher Scientific UK) with 10% (v/v) heat-inactivated foetal bovine serum (FBS), penicillin (10^2^ IU.ml-^1^), streptomycin (100 μg.ml-^1^) and 2 mM l-glutamine.

### Adhesion assay

EA. hy926 were adjusted to 10x10^4^cell/ml and cultured on coverslips for 3–4 days till 70% confluence. Then, they were stimulated with 25 ng/ml of TNFα (PeproTech EC Ltd (London, UK) or with TNFα and free fatty acids, FFA (oleic and palmitic acid, 2:1, 500 μM) (Sigma-Aldrich Company Ltd. (Dorset, UK) overnight. The following day, blood was withdrawn and stimulated fresh with LPS or heat-killed bacteria. Endothelial cell monolayer was washed with PBS and incubated with whole stimulated blood (to analyse inflammatory markers and for light microscopic analysis) or red blood cell (RBC)-lysed stimulated blood (for SEM and TEM analyses) for 1 h at 37 °C. PBS contained calcium and magnesium as necessary cations in the activation of integrins as part of cell adhesion. The supernatant was analysed for inflammatory markers. Endothelial monolayer was washed again to get rid of RBCs and unbound cells, and the adherent complexes were analysed further by SEM, TEM and light microscopy. All qualitative measurements were assessed independently by a second observer.

In blockade experiments, activated endothelial cells were pre-treated before addition of HKK-stimulated whole blood as follows: with culture medium (control), anti-P-selectin isotype, anti-P-selectin antibody (clones 11711 and 9E1, respectively, R&D Systems, Abingdon, UK) (at 10 µg/ml, 30 min after TNFα and FFA stimulation) or angiopoietin 2 ligand trap termed R3, prepared as previously described [32] (10 µg/ml, overnight together with TNFα and FFA stimulation), and then processed as above.

### Statistical analysis

All results are given as mean ± standard error of the mean (SEM). Significance of changes was assessed by Kruskal–Wallis test and post hoc testing by Dunn’s multiple comparisons test. Flow cytometric data were analysed by ANOVA and Tukey’s multiple comparisons test as they follow Gaussian distribution. The TNFα level of J774 cell line stimulated with or without LPS was analysed by Mann–Whitney test. GraphPad Prism 7 software was used for all analyses. A value *p* < 0.05 was considered statistically significant in all cases.

## Results

### Analysis of inflammatory platelet–granulocyte aggregates in a whole blood stimulation assay using a single PAMP

**Fig. 1:**
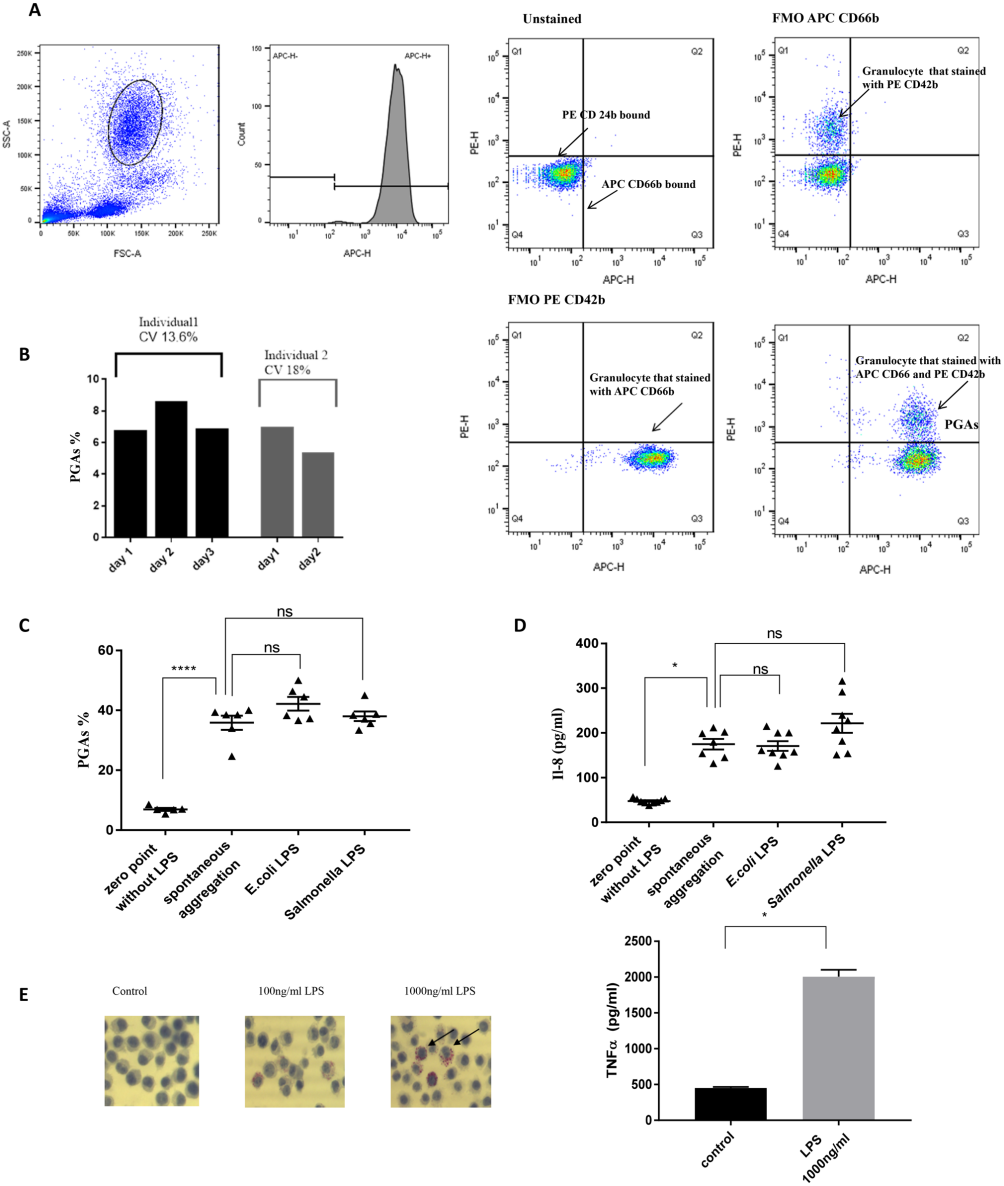
Inability of whole blood LPS stimulation to enhance platelet granulocyte aggregate formation over spontaneous aggregation. **a** Detection of platelet granulocyte aggregates by flow cytometry. The granulocyte population was clearly identified in whole blood based on light scatter characteristic, size and granularity. Approximately 99% of the cells in granulocyte gate were positive for the granulocyte marker CD66. From this granulocyte gate, events that stained positively for both CD66 (APC conjugate) and platelet marker CD42b (PE conjugate) were identified as platelet granulocyte aggregates, PGA. **b** Reproducibility of flow cytometric analysis of platelet granulocyte aggregates, PGA, by interassay test and intra-assay test. Interassay precision is expressed as coefficient of variation which is calculated by division of standard deviation by the mean and multiplied by 100, 13.6% for individual 1 and 18% for individual 2. Intra-assay precision is expressed as the average of the coefficient of variation, 15.8. **c** Summative presentation of six independent experiments, showing the percentages of PGAs induced by incubation with and without LPS (1000 ng/ml) at different conditions. **d** IL-8 level at different conditions of stimulation. **e** Ability of LPS, 1000 ng/ml, to induce inflammatory response in J774 mouse macrophages cell line expressed as induction of intracellular inclusions which were stained with Oil Red O and TNFα level detected in culture supernatants. Data are presented as mean ± SEM. Significance of changes was assessed by analysis of variance (ANOVA) and Tukey’s multiple comparisons test **c**, Kruskal–Wallis test and Dunn’s multiple comparisons test **d**, *n* = 5 or Mann–Whitney test, **e**, *n* = 3. Changes were considered significant if *p* value was < 0.05. FMO, fluorescence minus one control

Incubation of unstimulated whole blood at 37 °C for 1 h induced a significant increase in platelet–granulocyte aggregate formation compared to baseline (zero point, defined as the percentage of aggregate formation in the immediately stained blood sample) (Fig. 1a). An optimised protocol of sample collection, preparation, fixation and flow cytometric analysis was followed strictly for each sample. The reproducibility of that method was evaluated in terms of interperson reproducibility and interday validation by calculating the coefficient of variation (CV) for detecting the double-positive population (Fig. 1b) and met the general criteria for flow cytometry assay precision with CV < 20% [33].

Unexpectedly, incubation of whole blood with *E. coli* 0111*: B4* LPS or *Salmonella enteritidis* LPS for 1 h for both EDTA- and hirudin-anticoagulated samples did not induce an increase in platelet granulocyte aggregate levels beyond that of unstimulated whole blood incubated for 1 h at 37 °C (Fig. 1c).

The ability of LPS to induce the platelet granulocyte aggregate formation in blood had been demonstrated in vitro [21, 34], but while most in vitro whole blood LPS stimulation studies used LPS at 1 µg/ml for 1 h, these two studies used LPS at various doses ranging from 0.5 to 10 µg/ml for different times 1, 4 and 16 h. After finding no significant increase in platelet granulocyte aggregates level over spontaneous aggregation when different types of LPS at 1 µg/ml for 1 h and different anticoagulants (hirudin and EDTA) were used, we wanted to find out whether different concentrations of LPS at different time points of incubation could lead to a significant increase in these aggregates. To do so, aliquots of hirudin-anticoagulated whole blood (1 ml) were incubated with different concentrations of *Salmonella* LPS (0 [control], 0.5, 1.0 and 10.0 μg/mL) for 1 h and 4 h. The samples were immune-labelled and analysed by flow cytometry as before. Different doses of *Salmonella* LPS did not increase the number of platelet granulocyte aggregates significantly beyond the spontaneous aggregation observed at baseline for 1 and 4 h stimulation (data not shown). There was no production of IL-8, a pro inflammatory cytokine released in response to inflammatory stimuli [35] as well as TNFα (data not shown) in hirudin-anticoagulated (Fig. 1d) or EDTA-anticoagulated (data not shown) whole blood in response to LPS, while the same dose used to stimulate a macrophage cell line produced an inflammatory cellular response in terms of lipid inclusion and TNFα level in the presence of 10% (v/v) foetal calf serum (Fig. 1e).

### Analysis of inflammatory platelet granulocyte aggregates in a whole blood stimulation assay using PAMP-rich stimulation

Having determined that quantitative analysis of platelet granulocyte aggregates formation in response to LPS using flow cytometry did not detect a significant increase over spontaneous aggregation in spite of the ability of LPS to induce inflammatory response in cell culture model, we wanted to investigate the possibility of LPS to induce platelet granulocyte aggregates that might be structurally different from spontaneously forming aggregates, which could make these difficult to be analysed by flow cytometry. In addition, we included sepsis-relevant pathogenic surfaces in our studies, namely *K. pneumoniae* and *S. aureus.*

**Fig. 2:**
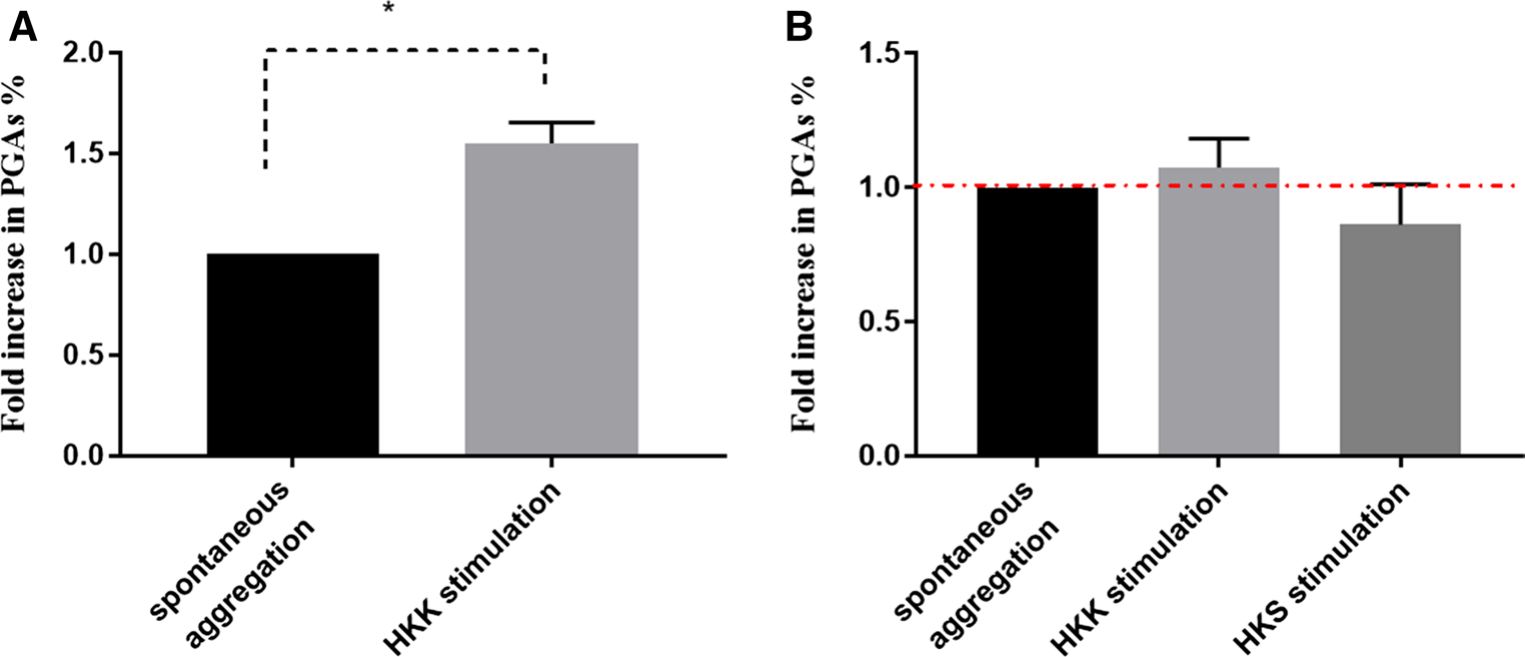
Platelet granulocyte aggregates induced by incubation of whole blood with and without heat-killed bacteria. PGAs were determined by flow cytometry as CD66^+^CD42b^+^ events and expressed as a fold increase in percentages of platelet granulocyte aggregates (PGAs) from total granulocytes population at different conditions. **a** Stimulation of EDTA-anticoagulated whole blood with HKK induces a significant increase in PGAs over spontaneous aggregation. **b** Stimulation of hirudin-anticoagulated whole blood with heat-killed *K.pneumoniae* (HKK) or heat-killed *S.aureus* (HKS) did not induce a significant increase in PGAs over spontaneous aggregation as detected by flow cytometry. Data are presented as mean ± SEM. Significance of changes was assessed by analysis of variance (ANOVA) and Tukey’s multiple comparisons test. **a***n* = 3 and **b***n* = 5. Changes were considered significant if *p* value was < 0.05

EDTA-anticoagulated whole blood was stimulated immediately with or without (control) heat-killed *K. pneumoniae*, HKK. The stimulated samples were incubated at 37 °C for 1 h. Then, aliquots of whole blood were stained with platelet- and granulocyte-specific markers as described above. Flow cytometric analysis of all samples obtained from two male and two female donors (different ages and ethnicity) shows a significant, 1.5–2-fold, increase in the platelet granulocyte aggregate level in the stimulated samples compared to the matched unstimulated samples (Fig. 2a). This is in good agreement with other studies which have shown nearly the same amount of platelet granulocyte aggregates increase that resulted from treatment of EDTA-anticoagulated whole blood with thrombin receptor activating peptide (TRAP, 0.3 μM) [36] or citrated whole blood with shiga toxin [34]. Having demonstrated that stimulation of EDTA-anticoagulated whole blood with HKK leads to a significant increase in platelet granulocyte aggregates formation, we now wanted to find out whether stimulation of whole blood with HKK and HKS as well but using hirudin as anticoagulant, that maintains availability of relevant cations, would also lead to an increase in platelet granulocyte aggregates. Multiple whole blood samples were collected into hirudin on different days. Each sample was divided into three aliquots and incubated immediately without any stimulus as a control or with HKK and HKS (10^6^ CFU/ml) separately at 37 °C for 1 h. Then, the samples were stained and processed for flow cytometric analysis. As shown in Fig. 2b, in comparison with the percentages of platelet granulocyte aggregates detected in unstimulated samples, there is surprisingly no significant increase in PGAs  % towards heat-killed *K. pneumoniae* or heat-killed *S. aureus*. It is possible that anticoagulation with hirudin—which inhibits coagulation by binding to activated thrombin, while EDTA chelates ions needed at the onset of coagulation—allows formation of aggregates of greater complexity. To address this, scanning electron microscopy (SEM) was used to study the morphology and the composition of inflammatory aggregate induced by LPS and HKK/HKS. Hirudin-anticoagulated whole blood samples taken from four healthy volunteers on different days were processed immediately as follows: each sample was incubated with *Salmonella* LPS (1000 ng/ml), HKK or HKS prepared from cultures at 10^6^ CFU/ml or left without stimulus as a control for 1 h at 37 °C, and processed in parallel for SEM.

In SEM images, leucocytes were identified based on their morphological features including folds, ruffles, projections and microvilli as well as their size, which ranged from 6 to 14 μm [37]. Although each white blood cell subtype (lymphocytes, monocytes, and granulocytes) under normal condition had different surface features recognised by SEM, there is overlap in the criteria, making the distinct identification of each subtype by SEM alone quite difficult [38]. In addition, one has to take into account that inflammatory stimulation of the cells of interest is likely to induce adaptive variation in their surface morphology that contributes in its own right to the difficulty of identifying leucocyte subtypes by SEM. That is why the platelet granulocyte aggregates will be named platelet-leucocyte aggregates hereafter. Platelets were reliably identified based on the previous observations of their surface morphological appearance which existed in two forms: an inactivated form with discoid shape and smooth surface (around 2 μm in diameter) and an activated disc or spherical shape with irregularly distributed long pseudopodia and protrusions [38, 39]. Some remaining red blood cells were identified by their general biconcave shape or as crenated erythrocytes, spherical with spicules, because of osmotic pressure changes during erythrolysis and SEM preparation [40].

**Fig. 3:**
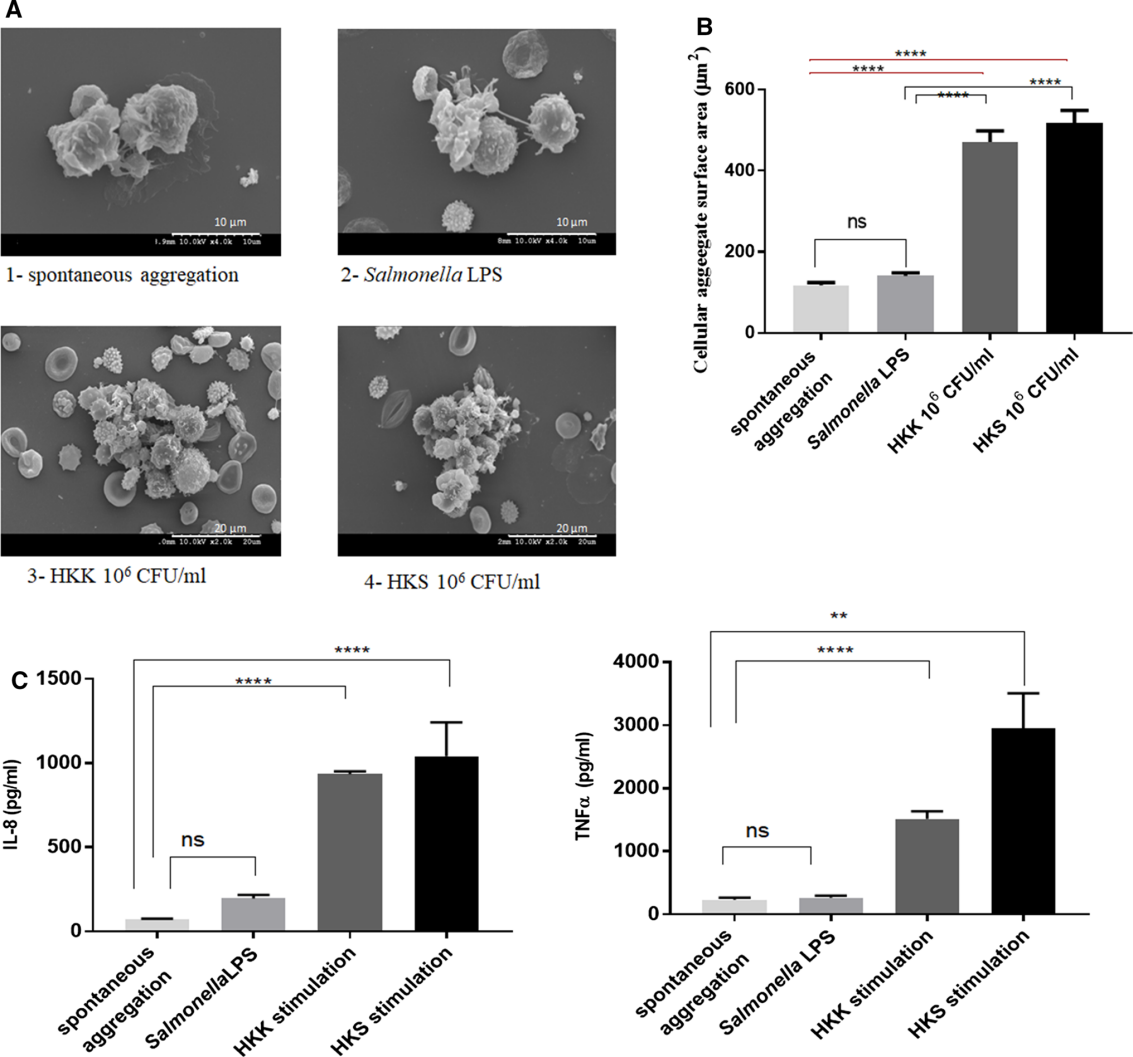
Comparative analysis of sepsis-relevant stimuli on platelet-leucocyte aggregate formation. Scanning electron microscopic analysis of cellular aggregate morphology and composition. **a**, **b** Representative images of cellular aggregates are shown from unstimulated sample (control) (1), after stimulation with *Salmonella* LPS (2), heat-killed *K. pneumoniae* (HKK) (3) or heat-killed *S. aureus* (4) (**a**). Semi-quantitative analysis was performed to assess the extent of cell aggregation in terms of its surface area (μm) using Image J software (**b**). **c** shows IL-8 and TNFα levels in plasma prepared from previous whole blood sample. Data are expressed as mean ± SEM and were analysed by means of Kruskal–Wallis test, followed Dunn’s multiple comparisons test between groups. Changes were considered significant if *p* value was < 0.05. Scale represents 10 (A1 and 2) or 20 μm (A3 and 4). SEM *n* = 64 images from *n* = 4 independent isolations per condition

In the unstimulated sample, most of the cellular aggregates were formed of one or two leucocytes surrounded by a few platelets and RBC and were found similar to the form of dominating aggregates induced by whole blood stimulation with LPS. HKK and HKS produced more complex aggregates (Fig. 3a), which occupied a significantly greater surface area compared to aggregates observed in unstimulated or LPS-stimulated samples (Fig. 3b). We conclude that the lack of detection of increased HKS/HKK-induced aggregates by flow cytometry in hirudin-anticoagulated blood (Fig. 2) was likely related to the complexity/size of the aggregates. The HKK- and HKS-induced aggregates were composed of many leucocytes, activated platelets, crenated erythrocytes and plenty of vesicles of less than 1 μm, which might be microparticles released by activated platelets, leucocytes and erythrocytes [41] (Fig. 3a). Consistently, significant increases in IL-8 and TNFα levels were found in HKK- and HKS-stimulated whole blood samples compared to unstimulated or LPS-stimulated samples (Fig. 3c).

### Adherence to endothelial cells of platelet-leucocyte aggregates formed in response to different stimuli under proinflammatory condition

The adhesion of inflammatory aggregates, induced with different stimuli, to TNFα-stimulated endothelium was evaluated using light microscopy and transmission electron microscopy (TEM).

**Fig. 4:**
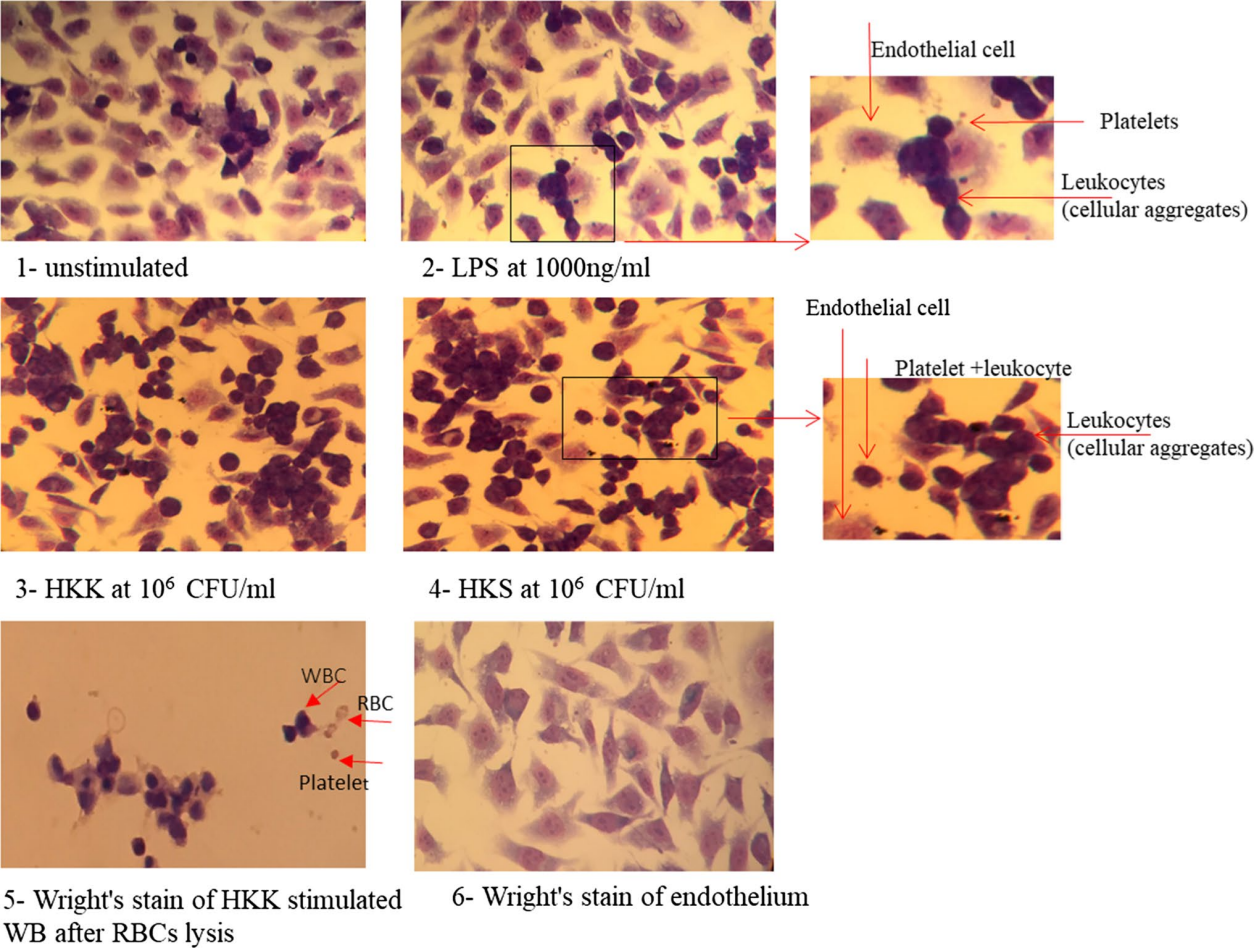
Light microscopic analysis of PLAs adherence to endothelium in response to different stimuli. Whole blood was left unstimulated (control) or stimulated with LPS (1000 ng/ml), heat-killed *K. pneumoniae* (HKK) or heat-killed *S. aureus* (HKS) and co-incubated with endothelium previously stimulated with TNFα (overnight). Samples were prepared for Wright’s stain analysis under light microscopy. Representative images of the adhesion model using unstimulated whole blood (1), LPS-stimulated (2), HKK-stimulated (3) or HKS-stimulated (4) whole blood (WB), allowing analysis of aggregates, their light microscopic composition (× 40 objective). HKK-stimulated whole blood after lysis (5) and TNFα-activated endothelium without blood (6) shown as controls

Light microscopic analysis demonstrated that a small number of cellular aggregates, formed spontaneously in unstimulated whole blood samples, adhered in a sporadic manner to endothelial cells (Fig. 4). A similar pattern of cellular aggregate adhesion was observed when LPS-stimulated whole blood samples were used. In contrast, when whole blood stimulated with heat-killed bacteria was used, large and complexed cellular aggregate frequently adhered on the endothelial layer. Further quantitative analyses were performed by counting the number of cellular aggregates per mm^2^ of endothelial layer and the number of discernible platelets and leucocytes involved in each cellular aggregate (Table 1). When the incubated blood on endothelium layer was unstimulated or stimulated with LPS, not only the number of cellular aggregates/mm^2^ but also the composition of the adhered aggregates, in terms of how many platelets and leucocytes were identified under microscopic examination to be involved in aggregate form, was comparable. However, a nearly twofold increase in the two parameters was observed when HKK or HKS was used as a stimulus to the whole blood, resulting in large complexes.

**Table 1:** Semi-quantitative light microscopic analysis of PLAs adherence to TNFα-stimulated endothelium in response to different stimuli

	Spontaneous aggregation	LPS	HKK	HKS
Number of cellular aggregates/mm^2^ (mean of *n* = 5)	16/mm^2^	15/mm^2^	34/mm^2^	27/mm^2^
Number of platelets/leucocytes in aggregates	1.6 leucocytes with 3.8 platelets (mean of *n* = 5)	1.9 leucocytes with 7.3 platelets (mean of *n* = 9)	2.75 leucocytes with 7.75 platelets (mean of *n* = 4)	3.1 leucocytes with 7.5 platelets (mean of *n* = 5)

**Fig. 5:**
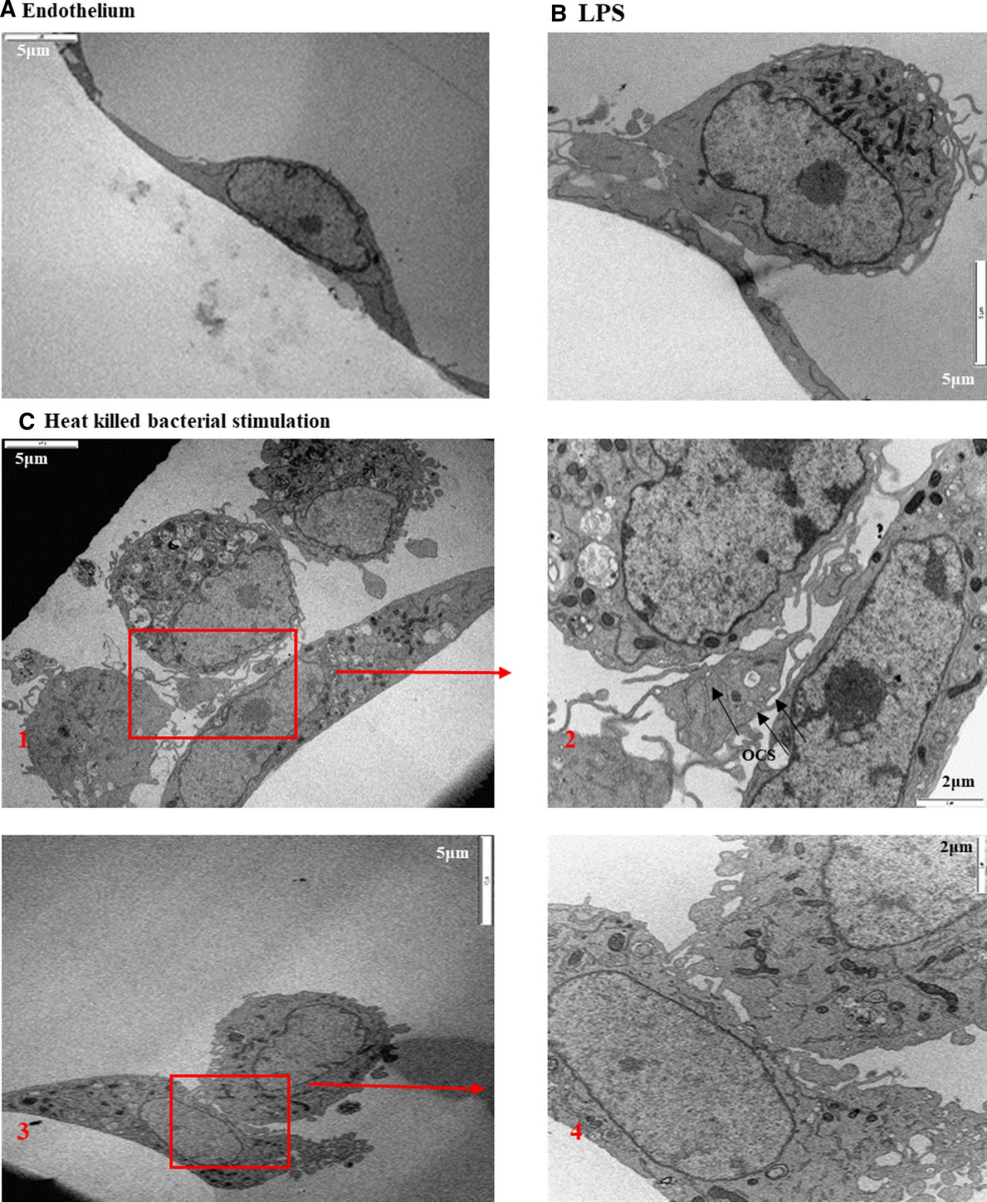
PLA aggregate adhesion to the endothelium in response to different stimuli. The transmission electron microscope micrograph shows a human endothelial monolayer treated with proinflammatory stimuli (TNFα) and incubated with human peripheral blood stimulated with different stimuli (LPS, HKK and HKS). **a** Monolayer of endothelium. **b** Representative image of adhered aggregate, preformed in response to whole blood LPS stimulation, **c** representative images of attached aggregates formed in response to heat-killed bacteria. OCS, open canalicular system. Scale (white bar at edge of image) represents 5 and 2 μm as indicated

TEM clearly showed the ultrastructural evidence of inflammatory cellular aggregate adhesion to activated endothelium monolayer and further confirmed the complex pattern of adhered aggregates detected earlier in this study (Fig. 5). Leucocyte and platelet can be identified based on their size and different cellular ultra-structures. Platelets were determined by their size (2–5 μm in diameter), absence of nuclei, open canalicular system and formation of filipodia [42], while leucocytes were mainly recognised by their diameter, which range from 8 to 10 μm, and nuclei [43].

**Fig. 6:**
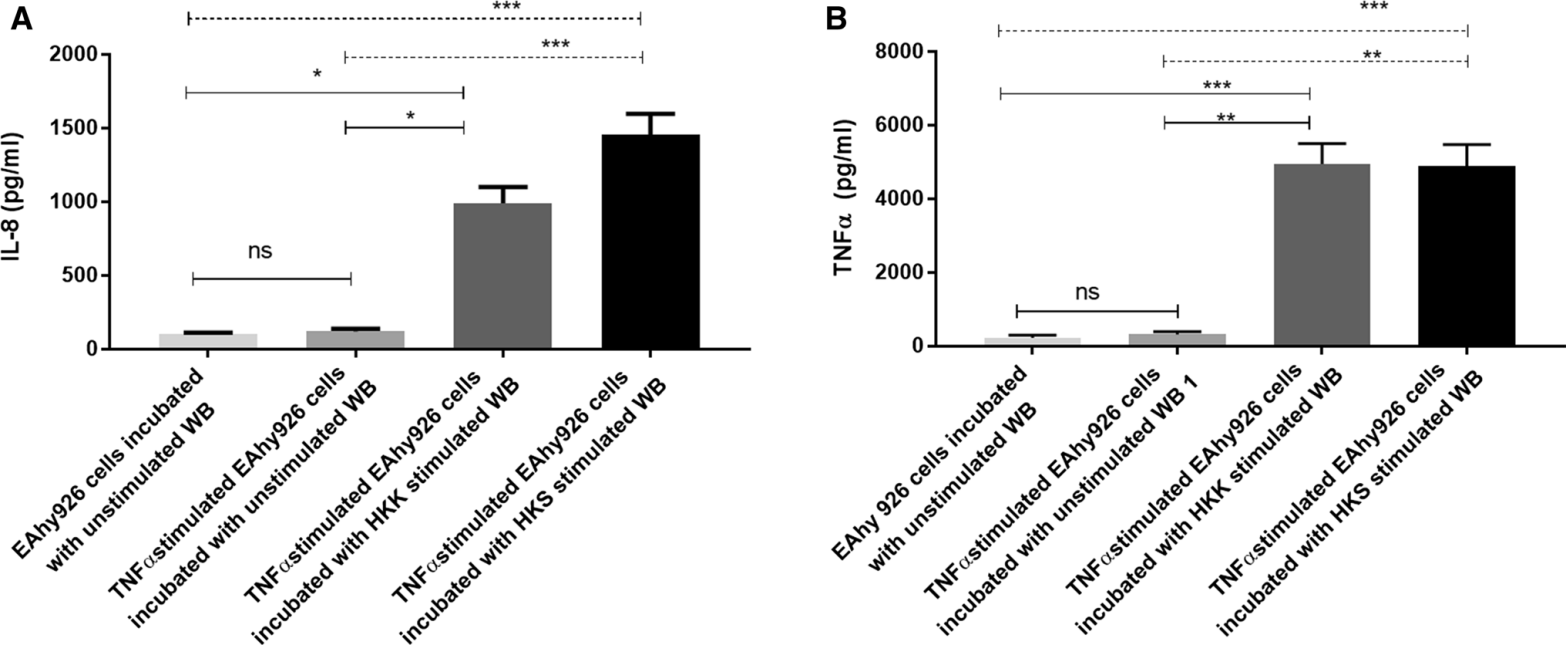
Analysis of inflammatory markers IL-8 and TNFα, under different conditions involving activated endothelial cells. Supernatants were analysed for IL-8 (**a**) and TNFα (**b**). *WB* whole blood. Data are expressed as mean ± SEM and were analysed by means of Kruskal–Wallis test, followed Dunn’s multiple comparisons test between groups. Changes were considered significant if *p* value was < 0.05. *n* = 4

In keeping with findings of a significant formation of aggregates after incubation with heat-killed bacteria-stimulated whole blood samples which adhere to activated endothelium, as shown by SEM, TEM and light microscopy, a significant increase in IL-8 and TNFα production in heat-killed bacteria-stimulated whole blood samples was detected after incubation with activated endothelium (Fig. 6).

### Effect of angiopoietin-2 ligand trap and P-selectin blocking antibody on inflammatory aggregate adherence to inflamed endothelium

**Fig. 7:**
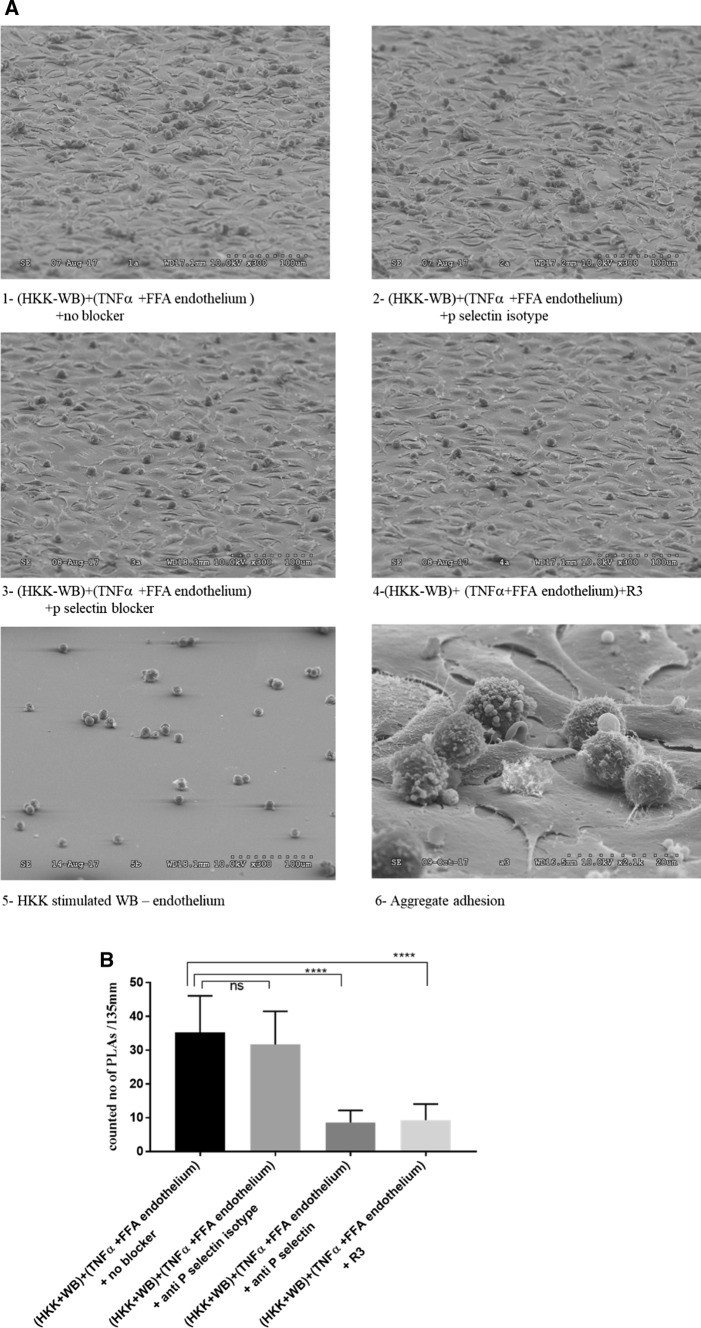
Blocking of inflammatory aggregates adherence to the activated endothelium by incubation with angiopoietin-2 ligand trap (R3) and P-selectin blocking antibody. **a** Tilted scanning electron microscope micrograph at 75° shows activated endothelial monolayer pre-treated with (1) culture medium (control), (2) anti-P-selectin isotype, (3) anti-P-selectin antibody, (4) the angiopoietin-2 ligand trap R3 and incubated with human peripheral blood stimulated with HKK, (5) SEM of HKK-stimulated blood shown as control and (6) SEM of aggregate adhesion at higher magnification (2.1 K). **b** Semi-quantitative analysis by counting the number of aggregate adhered to endothelium layer per 135 mm to assess the blocking effect using Image J software. Data are expressed as mean ± SEM and were analysed by means of Kruskal–Wallis test, followed Dunn’s multiple comparisons test between groups. Changes were considered significant if *p* value was < 0.05. Scale (line of grey dots at bottom right) represents 100 μm (1, 2, 3, 4, 5) and 20 μm (6). SEM *n* = 30 images from *n* = 3 independent isolations per condition

We have previously shown that addition of an evolved Tie2 ectodomain with selectivity for angiopoietin-2, termed R3 [32], results in blocking of angiopoietin-2 signalling, thereby promoting vascular protective signalling by angiopoietin-1, an alternative ligand for the endothelial cell receptor tyrosine kinase Tie2 [44–46]. The ability of R3 or a functional P-selectin antibody to block the adherence of inflammatory aggregate, formed in response to HKK stimulation, to endothelial cells incubated with TNFα and FFA was more accurately evaluated using SEM rather than immunofluorescence, which bore technical limitations (e.g. interference of immunolabelling with aggregation phenotype, cell-specific variation in cell membrane labelling using Vybrant™ DiO Cell-Labeling Solution, Thermo Fisher Scientific, Loughborough, UK; data not shown). Treatment of activated endothelial cell with R3 at (10 µg/ml, overnight) or anti-P-selectin (10 µg/ml, 30 min) prior to incubation with HKK-stimulated whole blood similarly reduced the inflammatory aggregate adhesion significantly compared to untreated background profile (Fig. 7).

## Discussion

In sepsis, there is extensive formation of inflammatory platelet-leucocyte aggregates that circulate and adhere to activated vascular endothelium. Platelet-leucocyte aggregates correlate with severity of disease. Adhesion of platelet-leucocyte aggregates to activated endothelial cells plays a significant role in sepsis pathogenesis. It leads to microvascular occlusion, slows the blood flow and activates the coagulation system preceding organ dysfunction and death [47]. Intravascular microscopic studies demonstrate that platelet neutrophil aggregation on activated endothelium is an important determinant of microvascular occlusion during inflammation [12].

Whole blood stimulation assays and flow cytometry have been widely used to study the formation of aggregates involving platelets and leucocytes as an in vitro approach to understand the acute inflammatory reaction in the bloodstream to the presence of PAMPs during septicaemia. Endotoxin, a wall component of gram-negative bacteria, is a stimulus relevant in the development of sepsis. PLAs have been analysed by flow cytometry using antibodies which bind to receptors expressed by platelets (such as CD41, CD42a, CD42b and CD61) and granulocytes (such as CD11b, CD16 and CD66) and gating for the double-positive events after compensation. However, this approach lacks robust methodology. For example, the extent of spontaneous formation of platelet-leucocyte aggregates ex vivo is often unclear. This highlights the need of a reliable in vitro model to investigate sepsis-relevant formation of platelet-leucocyte aggregates and their adhesion to activated endothelial cells.

In this study, a whole blood stimulation assay was used as in vitro approach to generate and analyse the formation of inflammatory aggregates involving platelets and leucocyte. It was seen as a more accurate model to develop greater understanding of the acute inflammatory reaction in the bloodstream to the presence of PAMPs during septicaemia for the following reason: in contrast to stimulation of isolated peripheral blood cells or peripheral blood cell culture, it provides a more physiological environment that allows a broader assessment of relevant effector molecules in plasma. It also avoids as much as possible artefactual activation of cells of interest [37, 48, 49].

The choice of anticoagulant was a key issue in this study. EDTA anticoagulates by chelating calcium ions which are necessary in the intrinsic and extrinsic pathway of the blood clotting cascade; hirudin inhibits thrombin specifically, thereby allowing initial activation of the cascade. While the extrinsic pathway is triggered by endothelial defect, the intrinsic pathway can be activated by pathogens [50]. In contrast to EDTA, citrate and heparin, hirudin allows a more accurate investigation of platelet-leucocyte interaction without interference with complement activity or depletion of divalent cations [51]. Compared to heparin, hirudin is favoured in the study of cell activation although it does not allow evaluation of the contribution of thrombin to the observed phenotype [52]. A large proportion of aggregate formation at baseline between leucocytes and platelets such as that observed in this study under static condition has previously been described, occurring in heparin- and citrate-anticoagulated blood. This aggregate formation could be reduced by incubation with fibrin polymerising inhibiting peptide such as GPRP and RGDS [34, 53, 54]. The spontaneous aggregate formation observed in our conditions (hirudin or EDTA), however, could not be blocked by addition of GPRP at the recommended dose of 5 mM (data not shown). Hirudin avoids reaction of fibrinogen to fibrin, so is as good a model as one can do in vitro to mimic the relative excess of procoagulant components (due to decreased anticoagulant pathways in vivo [55]). It follows that our system does include, in the presence of hirudin, a possible activity of fibrinogen binding to CD11c/CD18 (leucocyte integrin p150,95 or complement receptor 4) and CD11b/CD18 in the adhesion of endothelial cells, leucocytes and platelets.

We initially set out to gauge whether the experimental design of comparing the LPS-stimulated blood sample stained after incubation with the immediately stained, unstimulated sample in favour of comparing the LPS-stimulated blood sample with the unstimulated blood sample stained at the same time, was significant for the interpretation of results.

Blood collected (by venepuncture) into hirudin or EDTA and immunolabelled within 10 min prior to any treatment showed on average ~ 11% ± 5% platelet granulocyte aggregates in the total granulocyte population (PGA/G) by flow cytometry. This is consistent with data from other laboratories [56]. These authors showed a baseline (i.e. zero time point) of  ~ 15.3% leucocyte-forming aggregates with platelets in untreated blood from 36 healthy volunteers. They concluded that these aggregates were already preformed in vivo because blocking antibodies did not induce a significant decrease in these aggregate levels in the absence of in vitro stimulation [56]. Another study reported similar baseline of circulating platelet-leucocyte aggregates (7 ± 4% PLA/L) among healthy controls [57]. Thus, the detected baseline can be used to reflect the in vivo state and enables further experiments to investigate the underlying mechanisms involved in their formation. Aggregate detection was significantly increased to ~ 37% ± 10 PGA/G when untreated blood was incubated at 37 °C for up to 60 min. The unexpected large proportion of aggregate formation at baseline between granulocytes and platelets observed in this study under static condition has not clearly been described in previous studies. One study observed that fixation of the blood or lysis of erythrocytes associated with multiple centrifugation and washing resulted in an artefactual increase by three- to fivefold of platelet-leucocyte aggregates detected in unfixed blood [56]. The reported PLAs fold increase agrees with that observed in the present study after 1-h incubation at 37 °C. The discrepancies between the data could be attributed to the differences in sample processing and measurement. However, after treatment with LPS, no significant increase in percentage PGA could be detected over time. This is inconsistent with most of current papers as they compare the effect of stimulation, requiring incubation condition, on % platelet granulocyte aggregates with unstimulated sample stained immediately at zero time points [20, 21]. However, there is one study where no significant increase in platelet-leucocyte aggregate level was demonstrated after stimulation of whole blood with *E.coli* LPS at various concentrations over a wide range of 0.1, 1.0 and 10 μg/ml [58]. Similar to the present study, the latter study used a control incubated at the same condition of LPS-stimulated sample and thus allowed an accurate analysis of the effect of LPS stimulation. The same results were observed after stimulation with different doses of LPS (0.5, 1 and 10 μg/ml) for 1- and 4-h incubation. In addition, these results were comparable with SEM results, which showed ultrastructural morphology of aggregate formation with similar calculated surface area, allowing it to be properly analysed by flow cytometry.

The LPS types used to induce the formation of platelet granulocyte aggregates were able to stimulate an inflammatory cell response in a mouse macrophage cell line in the presence of 10% foetal calf serum in terms of TNFα production and lipid inclusions. These results are at variance with the ability of LPS to induce an inflammatory response in a whole blood assay that showed no significant difference in IL-8 concentration when EDTA- or hirudin-anticoagulated whole blood was stimulated with LPS from *Salmonella* or *E. coli*.

The fact that no significant effect of LPS on the formation of platelet granulocyte/leucocyte aggregates in a whole blood stimulation assay was detected by quantitative (flow cytometry) and qualitative methods (SEM) could be due to the unavailability of an appropriate dose of LPS to induce inflammation, resulting from the ability of the plasma compartment to basically neutralise its effect. For example, plasma lipoproteins, high-density lipoprotein, low-density lipoprotein, very low density lipoprotein and chylomicron remnants have been shown to neutralise the endotoxin effect by binding LPS [59, 60]. Additionally, several studies demonstrated the ability of antimicrobial peptides and many biological molecules with poly cationic structure such as procalcitonin, a precursor of calcitonin hormone, to neutralise the LPS effect [61]. Various plasma factors and membrane receptors are found to bind LPS and neutralise its effect. Lactoferrin, apolipoprotein A-1, apolipoprotein B, soluble CD14 (sCD14) and receptors expressed on macrophages, scavenger receptors, CD11b/CD18 receptors are involved in LPS detoxification [62].We have previously identified limitations in using endotoxin as a model to study the hyperacute phase of shock [63].

The finding that incubation of the LPS unstimulated sample for the same time as parallel samples that are stimulated with LPS (1 h) led to considerable aggregate formation means that the baseline with which the LPS-stimulated samples need to be compared was significantly elevated over the immediately stained whole blood sample. This observation significantly impacts on the conclusions drawn from this in vitro model of whole blood stimulation.

Our data imply that, in contrast to the qualitative analysis by SEM, the quantitative analysis using flow cytometry may not be an accurate way to assess formation of platelet-leucocyte aggregates in response to inflammatory stimulation of a whole blood assay for several reasons. Importantly, in this study, the size of inflammatory aggregate was found to affect the accuracy of flow cytometric analysis. SEM results show that most of the aggregate formed in response to HKK or HKS stimulation of hirudin-anticoagulated whole blood was too large (with a mean surface area of 470.61 and 518.39 μm^2^, respectively) to be detected by flow cytometry, because the internal diameter of flow cell being used by modern flow cytometers usually ranges from 50 to 250 μm [64]. Therefore, the large particles with sizes more than 250 μm are not analysable by flow cytometry. Thus, detecting a significant increase in aggregate formation after HKK stimulation when EDTA-anticoagulated whole blood was used in contrast to hirudin when no increase was detected, appears to be strongly related to the effect of EDTA on the cellular interaction. This is because a considerable component of divalent cation platelet-leucocyte dependent interaction and complement related effects on propagation of this interaction is excluded that leads to underestimation of the actual aggregate with less complexity allowing them to be analysed using flow cytometry. Another important factor that affects accuracy of flow cytometric analysis of inflammatory aggregate formation is the morphology and the relative composition of the aggregate. In SEM analysis, different forms of aggregates, which can be analysed by flow cytometry in regard to their size, were found. Aggregates composed of two or more leucocytes (which could be any mix of granulocyte, monocyte or lymphocyte) and plenty of platelets at different ratios which are interpreted by flow cytometry as a single event as one platelet with one granulocyte. Some globular structure with corresponding size of granulocyte was completely covered by platelets which could mask the surface expression of granulocyte marker and be interpreted as single platelet by flow cytometry.

Platelet–leucocyte interaction induces a correlative stimulation of both platelet and leucocyte that results in modulating leucocyte function to clear and limit the spread of infection within the circulation or/and tissue. Leucocyte interaction with platelet facilitates its adhesion to endothelium/transmigration and infiltration locally to the site of inflammation and recruitment of more leucocytes [65, 66]. In sepsis, this process is significantly enhanced due to uncontrolled inflammation and leads to endothelium incompetency. Excessive leucocyte infiltration leads to hyperinflammation and organ damage. Therefore, endothelium was treated either with anti-P-selectin or R3. Current strategies in clinical trials target the endothelium with the aim to restore its competence [55] and thereby reduce microvascular occlusion, hypoperfusion and activation of the coagulation system preceding organ dysfunction and death.

Angiopoietin 2 levels in septic patients are strongly associated with elevated markers of endothelial inflammation [67]. Furthermore, angiopoietin-2 has been shown to be required for upregulation of leucocyte adhesion molecules on endothelium by TNFα and other inflammatory activators [68]. Aggregate formation is dependent to a large extent on the engagement of P-selectin expressed on both platelets and endothelium and PSGL-1 on leucocytes. The successful blockade of adhesion and formation of aggregates, respectively, shows that our design provides a suitable, pathophysiologically relevant, model, with which to study ex vivo cell interactions in the acute phase of septic inflammation. Importantly, blockades were successful in the presence of FFA, an additional inflammatory stimulus present as danger associated molecular pattern, in sepsis and hypoperfusion [69, 70].

All in all, our findings clearly indicate that endotoxin from gram-negative bacteria is not a sufficient stimulus to provoke sepsis-relevant platelet-leucocyte aggregates in vitro. Heat-killed bacteria are preferred in the studies of whole blood inflammatory cellular aggregates under simulated physiological conditions, i.e. at the time point of stimulation, presence of divalent cations levels and intact complement activity.

Therefore, to quantify the interference of blockers on adherence of platelet-leucocyte aggregates induced using whole blood stimulations, we recommend using a heat-killed clinical isolate, which provides a wide range of pathogen-associated molecular patterns, in order to produce a model of acute cellular interactions in the fluid phase, which are relevant in the initial phase of sepsis.
